# Regulatory T cell-like response to SARS-CoV-2 in Jamaican fruit bats (*Artibeus jamaicensis*) transduced with human ACE2

**DOI:** 10.1371/journal.ppat.1011728

**Published:** 2023-10-19

**Authors:** Bradly Burke, Savannah M. Rocha, Shijun Zhan, Miles Eckley, Clara Reasoner, Amin Addetia, Juliette Lewis, Anna Fagre, Phillida A. Charley, Juergen A. Richt, Susan R. Weiss, Ronald B. Tjalkens, David Veesler, Tawfik Aboellail, Tony Schountz

**Affiliations:** 1 Department of Microbiology, Immunology and Pathology, College of Veterinary Medicine, Colorado State University, Fort Collins, Colorado, United States of America; 2 Department of Environmental and Radiological Health Sciences, College of Veterinary Medicine, Colorado State University, Fort Collins, Colorado, United States of America; 3 Department of Biochemistry, University of Washington, Seattle, Washington, United States of America; 4 Diagnostic Medicine/Pathobiology, Center of Excellence for Emerging and Zoonotic Animal Diseases, College of Veterinary Medicine, Kansas State University, Manhattan, Kansas, United States of America; 5 Department of Microbiology, Perelman School of Medicine, University of Pennsylvania, Philadelphia, Pennsylvania, United States of America; Icahn School of Medicine at Mount Sinai Department of Microbiology, UNITED STATES

## Abstract

Insectivorous Old World horseshoe bats (*Rhinolophus* spp.) are the likely source of the ancestral SARS-CoV-2 prior to its spillover into humans and causing the COVID-19 pandemic. Natural coronavirus infections of bats appear to be principally confined to the intestines, suggesting fecal-oral transmission; however, little is known about the biology of SARS-related coronaviruses in bats. Previous experimental challenges of Egyptian fruit bats (*Rousettus aegyptiacus*) resulted in limited infection restricted to the respiratory tract, whereas insectivorous North American big brown bats (*Eptesicus fuscus*) showed no evidence of infection. In the present study, we challenged Jamaican fruit bats (*Artibeus jamaicensis*) with SARS-CoV-2 to determine their susceptibility. Infection was confined to the intestine for only a few days with prominent viral nucleocapsid antigen in epithelial cells, and mononuclear cells of the lamina propria and Peyer’s patches, but with no evidence of infection of other tissues; none of the bats showed visible signs of disease or seroconverted. Expression levels of ACE2 were low in the lungs, which may account for the lack of pulmonary infection. Bats were then intranasally inoculated with a replication-defective adenovirus encoding human ACE2 and 5 days later challenged with SARS-CoV-2. Viral antigen was prominent in lungs for up to 14 days, with loss of pulmonary cellularity during this time; however, the bats did not exhibit weight loss or visible signs of disease. From day 7, bats had low to moderate IgG antibody titers to spike protein by ELISA, and one bat on day 10 had low-titer neutralizing antibodies. CD4^+^ helper T cells became activated upon ex vivo recall stimulation with SARS-CoV-2 nucleocapsid peptide library and exhibited elevated mRNA expression of the regulatory T cell cytokines interleukin-10 and transforming growth factor-β, which may have limited inflammatory pathology. Collectively, these data show that Jamaican fruit bats are poorly susceptible to SARS-CoV-2 but that expression of human ACE2 in their lungs leads to robust infection and an adaptive immune response with low-titer antibodies and a regulatory T cell-like response that may explain the lack of prominent inflammation in the lungs. This model will allow for insight of how SARS-CoV-2 infects bats and how bat innate and adaptive immune responses engage the virus without overt clinical disease.

## Author summary

Bats are reservoir hosts of many viruses that infect humans, yet little is known about how they host these viruses, principally because of a lack of relevant and susceptible bat experimental infection models. Although SARS-CoV-2 originated in bats, no robust infection models of bats have been established. We determined that Jamaican fruit bats are poorly susceptible to SARS-CoV-2; however, their lungs can be transduced with human ACE2, which renders them susceptible to the virus. Despite robust infection of the lungs and diminishment of pulmonary cellularity, the bats showed no overt signs of disease and cleared the infection after two weeks. Despite clearance of infection, only low-titer antibody responses occurred and only a single bat produced neutralizing antibody, consistent with other experimental infection studies of bats. Assessment of the CD4^+^ helper T cell response showed that activated cells expressed the regulatory T cell cytokines IL-10 and TGFβ that may have tempered pulmonary inflammation.

## Introduction

The ancestral severe acute respiratory syndrome coronavirus 2 (SARS-CoV-2), in all likelihood, originated in insectivorous bats prior to spillover to humans through one or more intermediate bridge hosts in live animal markets in Wuhan, China [1–4]. SARS-CoV-2 is a sarbecovirus (genus *Betacoronavirus*, subgenus *Sarbecovirus*) that uses angiotensin converting enzyme 2 (ACE2) as a cellular entry receptor in humans. Although hundreds of distinct sarbecovirus sequences have been detected in bats in Asia and Europe, principally in horseshoe bats (*Rhinolophus* spp.) [3,5–11], only some of these viruses can use ACE2 as an entry receptor [12].

Field studies of natural coronavirus infections of bats show that infections are localized to the gastrointestinal tract with likely shedding through feces [13], suggesting maintenance of virus in natural bat populations is via fecal-oral transmission [1,5–7]. However, few studies have experimentally examined coronavirus infections in bats, and those that have relied exclusively on surrogate models. Experimental challenge determined that Jamaican fruit bats (*Artibeus jamaicensis*), one of the most abundant and largest bats in the Americas, are susceptible to the merbecovirus, Middle East respiratory syndrome coronavirus (MERS-CoV) [14]. Viral antigen and RNA were detected in several organs up to 14 days post challenge, and viral RNA was detected in oral and rectal swabs up to 9 days post challenge. Despite clear evidence of infection, no conspicuous disease occurred, only one bat seroconverted, and they remained healthy. Inoculation of Jamaican fruit bat primary kidney cells (Ajk cells) led to robust virus replication and cytopathic effect, with destruction of the Ajk cell monolayer. MERS-CoV likely emerged from insectivorous bats in Africa [15–17] that used dromedary camels (*Camelus dromedarius*), which then became a secondary reservoir host species [18], as a bridge to humans.

Considering the wide species tropism of SARS-CoV-2, which includes mustelids, cervids, felines, canines and cricetid rodents, including Syrian hamsters and North American deer mice [19–25], it might be expected that SARS-CoV-2 can readily infect a variety of bat species. A significant limitation for conducting meaningful studies on the biology of bat-borne viruses is the lack of relevant captive breeding colonies of specific pathogen-free (SPF) bats. A few established colonies of SPF fruit bats and nectarivorous bats are available for infectious disease studies [26–28]; however, it is substantially more difficult to establish breeding colonies of insectivorous bats because of the need for live insect species that are often not natural food sources of the bats. Thus, insectivorous bats are typically captured and used for one-off experimental infection studies. Despite these limitations, previous work using Egyptian fruit bats (*Rousettus aegyptiacus*), and insectivorous big brown (*Eptesicus fuscus*) and Brazilian/Mexican free-tailed (*Tadarida brasiliensis*) bats have examined susceptibility to SARS-CoV-2 infections [29–32]. Although Egyptian fruit bats were moderately susceptible without disease, with detection of virus in the respiratory tract for several days and transmission to one other bat, big brown bats were not susceptible. There is conflicting evidence of susceptibility of Brazilian free-tailed bats; one study suggested they are not susceptible [31], whereas another suggested low susceptibility [32]. Thus, it is clear host specificity of SARS-CoV-2 infection cannot be determined simply by taxonomic assignment of a given mammalian species. Without closed, SPF breeding colonies of horseshoe bats and their sarbecoviruses, surrogate animal models could provide information about how these viruses behave in bats.

Because we have had a closed SPF breeding colony of Jamaican fruit bats, a New World bat species, we sought to determine its susceptibility to SARS-CoV-2. After determining that infection was limited to the intestines for only a few days, we then used an adenovirus vector to transduce cells of the lungs of bats with human ACE2 to determine infection and host response, including helper T cells, that can be compared to human COVID-19 infections. Although surrogate infection models often do not recapitulate human or reservoir infections, they permit examination of similarities and differences between humans and animal models that may be useful for understanding virus biology in other species. Considering the paucity of infection studies performed in bats, the use of human gene transduction performed herein permits a better understanding of bat responses to sarbecovirus infection.

## Results

### SARS-CoV-2 causes restricted infection of Jamaican fruit bats that is confined to the small intestine

**Fig 1:**
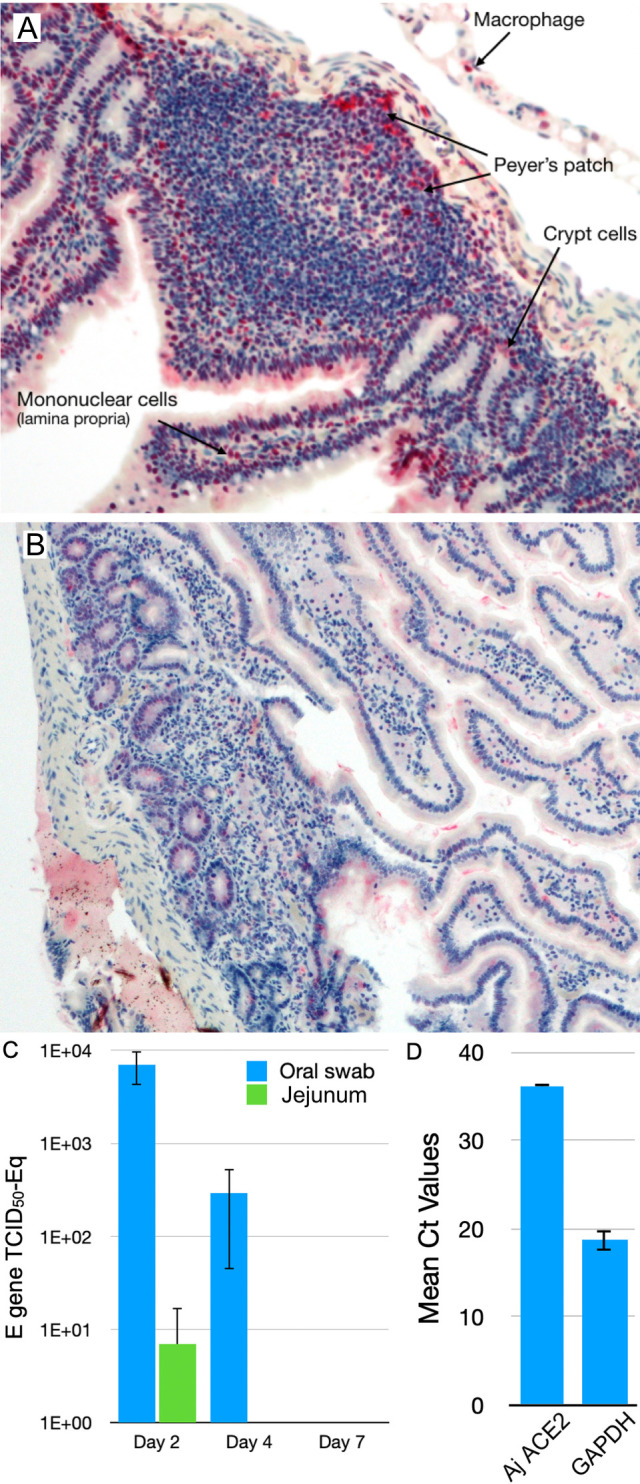
Immunohistochemistry of intestines of Jamaican fruit bats challenged with SARS-CoV-2. Eighteen bats were inoculated with SARS-CoV-2 WA1 and groups of 3 bats euthanized on days 2, 4, 7, 10, 14 and 21. On day 2, 3 naïve contact bats were added to the cage of inoculated bats that were euthanized on day 21, and 3 negative control bats were also euthanized on day 21 **A**. Two days after challenge, but not thereafter, antigen was detected in mononuclear cells of the lamina propria and periphery of Peyer’s patches where macrophages are typically found. Crypt cells also were infected. **B**. Unchallenged control bat intestine showed no antigen staining. **C**. SARS-CoV-2 E gene vRNA was detected only on days 2 and 4 in oral swabs, and only on day 2 in jejunum. **D**. Endogenous ACE2 expression in lungs of Jamaican fruit bat appears to be low and may account for the lack of detectable virus in lungs. No contact transmission was detected by qPCR of oral and rectal swab samples or viral antigen in tissues.

Jamaican fruit bats intranasally inoculated with SARS-CoV-2 WA1 were serially euthanized on days 2, 4, 7, 10, 14 and 21. No visible signs of disease and no weight loss occurred. However, viral antigen was readily detected in intestinal sections on day 2 (Fig 1A) but not day 4 or thereafter. Antigen was particularly prominent in the periphery of Peyer’s patches that are typically populated with macrophages, mononuclear cells of the lamina propria, and intestinal crypts. No antigen was detected in intestinal sections of unchallenged bats (Fig 1B). Oral swabs, but not rectal swabs, from bats were positive for viral RNA (vRNA) on days 2 and 4 post challenge (Fig 1C), but negative thereafter. Other organs were examined but no viral antigen was detected; nasal passages, brain, stomach, lung, heart, kidney, liver, spleen, gall bladder, pancreas. Despite clear evidence of antigen staining in intestines, both ELISA to recombinant nucleocapsid antigen and virus neutralization assay were negative for antibodies. To determine why virus was not detected in the lungs, we examined ACE2 gene expression levels and found that it was very low (Fig 1D), suggesting this may be a restriction factor for infection in the lungs.

### Defective adenovirus encoding human ACE2 transduces Jamaican fruit bat cells

**Fig 2:**
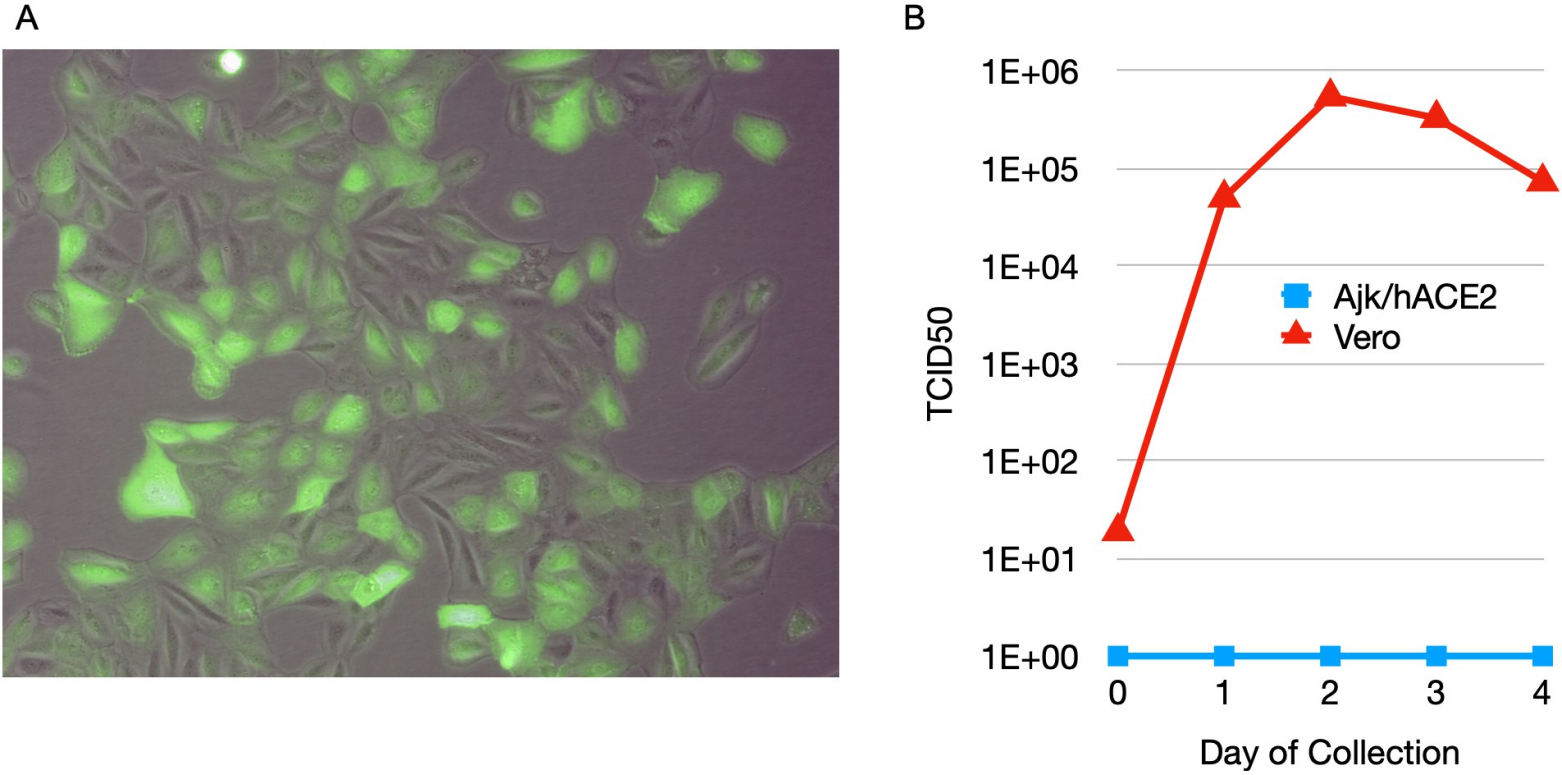
Adenovirus serotype 5 encoding human ACE2 (Ad5/hACE2) transduces Jamaican fruit bat cells in vitro. **A.** Transduction of Jamaican fruit bat primary kidney epithelial (Ajk) cells with Ad5/hACE2/eGFP. Jamaican fruit bat primary kidney cells were inoculated with 10 MOI of Ad5/hACE2/eGFP for 1 hour, washed and incubated in fresh medium for 4 days. Most, but not all, cells exhibited fluorescence, suggesting a heterogeneous population with some susceptible and some nonsusceptible cells. **B**. Ad5/hACE2 transduced Jamaican fruit bat kidney cells are not productively infected with SARS-CoV-2. Ajk/hACE2 cells (blue) and Vero cells (red) were inoculated with 0.1 MOI of SARS-CoV-2 WA1 for 1 hour, washed and cultured for 4 days. Supernatants were collected each day and titrated on Vero E6 cells to determine virus titers. Despite evidence of human ACE2 expression on Ajk cells, inoculation failed to produce infectious virus, suggesting the cells were not permissive.

A replication-defective adenovirus serotype 5 that encodes human ACE2 (Ad5/hACE2) has been used to study SARS-CoV-1 and SARS-CoV-2 infections of nonsusceptible laboratory mice [33,34]. Examination of the mouse receptor for Ad5, the coxsackievirus and adenovirus receptor (CAR), revealed that it has 85% identity and 92% similarity to Jamaican fruit bat CAR, suggesting that Jamaican fruit bats may be susceptible to Ad5. To test this hypothesis, we inoculated primary Jamaican fruit bat kidney (Ajk) cells with Ad5/hACE that also encodes eGFP and examined the cells daily. By 24 hours, florescence was observed and it was maximal on days 2 through 6 when the study was terminated (Fig 2A). Ajk cells, transduced for 48 hours, and Vero E6 cells (positive control) were inoculated with 0.1 MOI of SARS-CoV-2 and supernatants were collected at 1 hour and daily for 4 days, followed by titration on Vero E6 cells. No increase in SARS-CoV-2 was detected from hACE2-transduced Ajk cells (Fig 2B), suggesting the cells were not permissive for SARS-CoV-2 replication.

### Bat lung cells transduced with human ACE2 are susceptible to SARS-CoV-2

**Fig 3:**
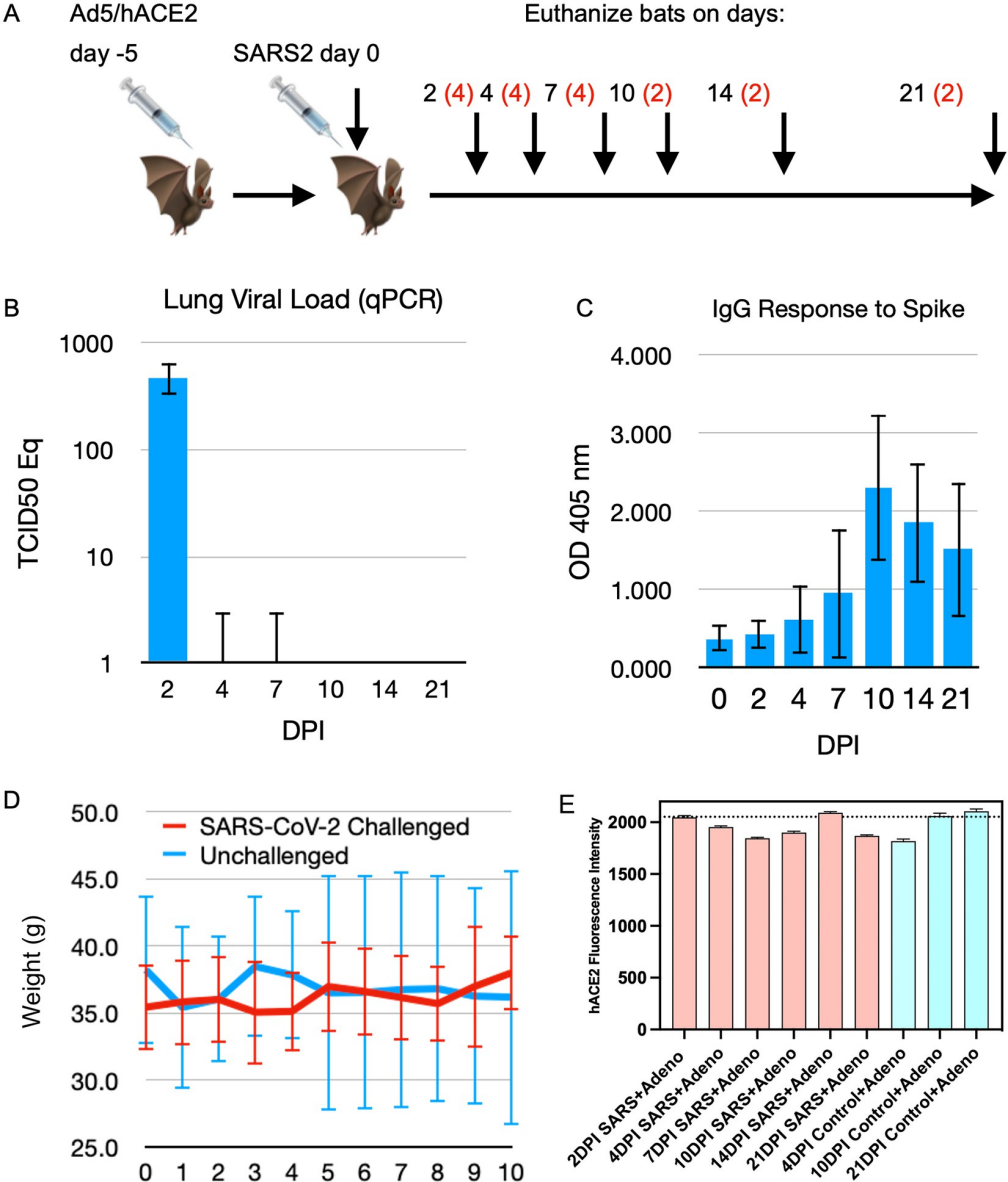
SARS-CoV-2 infection of Jamaican fruit bat lungs transduced with hACE2. Eighteen bats were transduced with human ACE2 followed by SARS-CoV-2 challenge 5 days later. Groups of bats were euthanized; 4 on days 2, 4, 7; 2 on days 10, 14, 21. **A.** Experimental design. Jamaican fruit bats were intranasally inoculated with Ad5/hACE2, then challenged with SARS-CoV-2 WA1. Groups of bats (n = red numerals) were euthanized at intervals and evaluated for infection. **B**. Viral RNA was detected in all bats on day 2 but only one bat on each of days 4 and 7. **C**. Serum antibody titers to recombinant spike antigen became elevated by day 4 in one bat, and all bats thereafter. **D**. Bats did not lose weight during the first ten days of the study, nor did they show other signs of disease. **E.** Human ACE2 expression levels were not statistically different between time points or between SARS-CoV-2-infected and uninfected bats.

To determine if lung cells of Jamaican fruit bats transduced with human ACE2 become susceptible to SARS-CoV-2, 21 bats were intranasally inoculated with 2.5x10^8^ pfu of Ad5/hACE2 and 5 days later 18 were challenged with SARS-CoV-2 (Fig 3A) while 3 served as negative controls. Groups of infected bats (four on days 2, 4, 7; two on days 10, 14, 21) were serially euthanized. On day 2, lungs of all four euthanized bats had detectable vRNA; however, on days 4 and 7 only one bat from each group had vRNA, and none of the lungs from bats thereafter had vRNA (Fig 3B). Serum antibody to recombinant spike was detected by ELISA in one bat on day 4, and all bats euthanized thereafter, with peak response on day 10 (Fig 3C). Only one bat, euthanized on day 10, had weak, but detectable, neutralizing antibody using a D614G VSV pseudotype assay (S1 Fig); this bat also had the highest O.D. value on the ELISA (2.957). No weight loss occurred in the bats (Fig 3D). Human ACE2 was detected in bats at a similar level through day 21 of the study (Fig 3E).

### SARS-CoV-2 lung pathology and localization in hACE2-transduced bats

**Fig 4:**
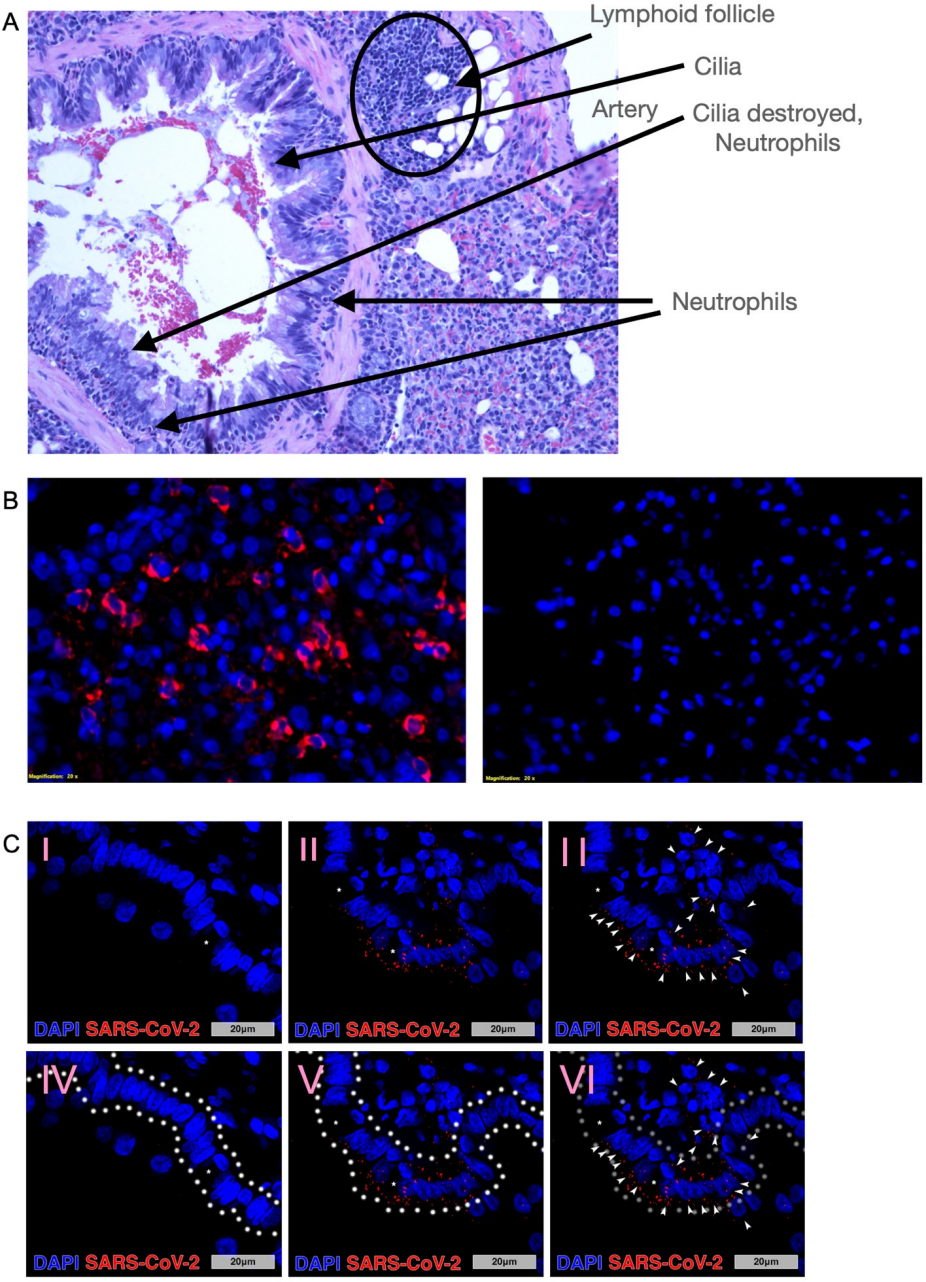
SARS-CoV-2 infection of Jamaican fruit bat lungs transduced with hACE2. **A.** Histopathology of Jamaican fruit bat lungs transduced with Ad5/hACE2 and infected with SARS-CoV-2, 4 DPI. Cilia destruction, neutrophil infiltration, and lymphoid expansion occurred. **B**. Fluorescent imaging of Ad5/hACE2 bat lung sections on day 7 infected with SARS-CoV-2 (left) or uninfected (right) showed perinuclear nucleocapsid antigen staining (red) (200x). **C.** Main stem bronchi (100x) in bats that received Ad5/hACE2 but no SARS-CoV-2 (I, IV) and bats that received Ad5/hACE2 followed by viral infection with SARS-CoV-2 at post-infection day 14 (14 DPI; II, III, V, VI). Asterisks identify goblet cells within the respiratory epithelium. Arrowheads identify cells that contain positive perinuclear SARS-CoV-2 nucleocapsid staining, that are composed of epithelium, goblet cells and cells of the lung parenchyma. White outlines highlight the respiratory epithelial layer (including goblet cells) of the bronchi for reference (IV, V, VI).

Infection of the lungs was principally localized around the main stem bronchia, with destruction of cilia corresponding with neutrophil infiltration, and lymphoid follicle formation (Fig 4A). Perinuclear SARS-CoV-2 nucleocapsid antigen was readily detected in the lungs of challenged bats for up to 14 days, whereas in unchallenged bats no antigen was detected (Fig 4B). On day 21, all bats were negative for nucleocapsid antigen, suggesting viral clearance. Epithelial, goblet and parenchymal cells had perinuclear staining for nucleocapsid antigen through day 14, suggesting these cells were susceptible to the Ad5 transduction of human ACE2 (Fig 4C).

### SARS-CoV-2 infection of bats expressing hACE2 leads to diminished cellular population within lung tissue

**Fig 5:**
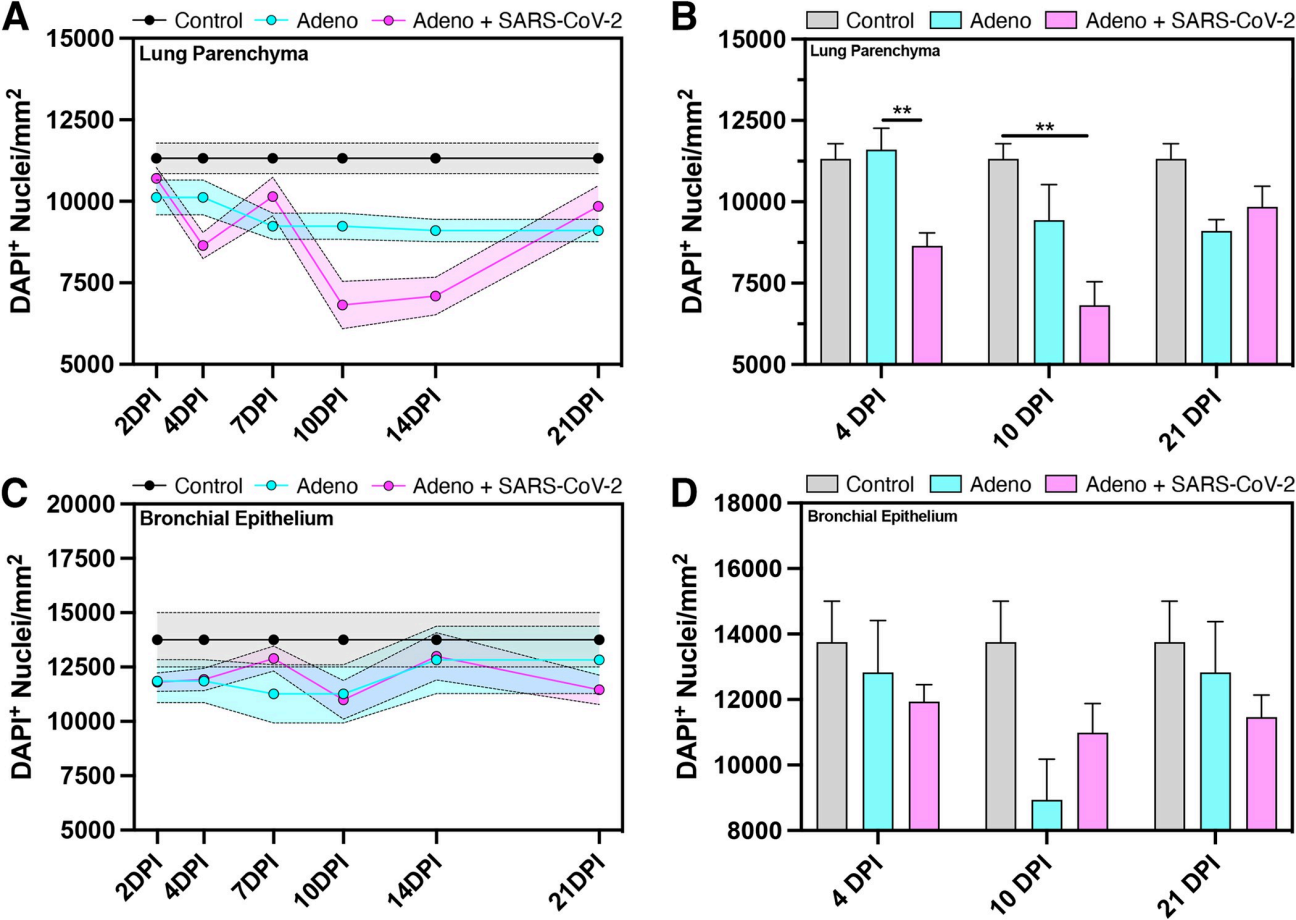
SARS-CoV-2 infection of hACE2 transduced bats leads to reduced numbers of cells in the lung parenchyma that is time-dependent. Time course analysis of total DAPI+ cellular populations of whole tissue montage images within the lung parenchyma (A, B) and bronchial epithelium (C, D) of no human ACE2 control bats (grey), hACE2 bats (blue), and hACE2 + SARS-CoV-2 bats (pink). n = 2–5 lung sections per animals/group. **p<0.01.

Total cellular quantification of whole lung tissue in control, Ad5/hACE2, and Ad5/hACE2/SARS-CoV-2 groups revealed temporal alterations in cellular populations that were dependent upon hACE2 expression and subsequent infection with SARS-CoV-2 (Fig 5). Time course analysis of DAPI^+^ nuclei within the lung parenchyma (Fig 5A) showed reduction in total cellular density over time where the greatest variance from the hACE2 group was observed at 4 DPI when compared to the Ad5/hACE2/SARS-CoV-2 group, and variance from control was detected in the Ad5/hACE2/SARS-CoV-2 group at 10 DPI (Fig 5A and 5B). Driving factors of variance between groupings was attributed to the following: the combination of hACE2, SARS-CoV-2, and time (p = 0.0468, F(4,28) = 2.768); hACE2 and SARS-CoV-2 (p = 0.0002, F(2,28) = 11.62) (Fig 5B). Time course assessment of DAPI^+^ bronchial epithelial cells (Fig 5C and 5D) revealed unremarkable changes where trending variance was observed from the combined analysis of hACE2 and SARS-CoV-2 infection (p = 0.0514, F(2,28) = 3.307).

### CD4^+^ T cells responded to SARS-CoV-2 in infected bats

CD4^+^ helper T cells control and orchestrate immune responses to viruses by secreting cytokines that dictate the activities of other cells. To determine if helper T cells responded to SARS-CoV2, we adopted an activation-induced marker (AIM) test to identify CD4^+^ T cell responses (32–34). This assay relies on increased expression of CD154 on helper T cells that occurs within hours of TCR engagement of MHC-II/peptide and its signal-to-noise ratio is augmented by blocking CD40 on the antigen presenting cells.

We screened several commercially-available anti-CD4 monoclonal antibodies (mAb) for cross reactivity; however, none were useful. Therefore, we generated a mAb (designated 1-D5) using a Jamaican fruit bat CD4 peptide (ENRKVSVVKTRQDRR) that was predicted to be extracellular and solvent-accessible, conjugated to KLH for mouse immunizations (S2 Fig). Based upon structural homology (S3 Fig), we determined that commercially available anti-mouse CD40 (Tonbo, clone FGK45) and anti-human CD154 (Tonbo, clone 5C8) monoclonal antibodies were cross reactive with Jamaican fruit bat orthologs (S4 Fig).

**Fig 6:**
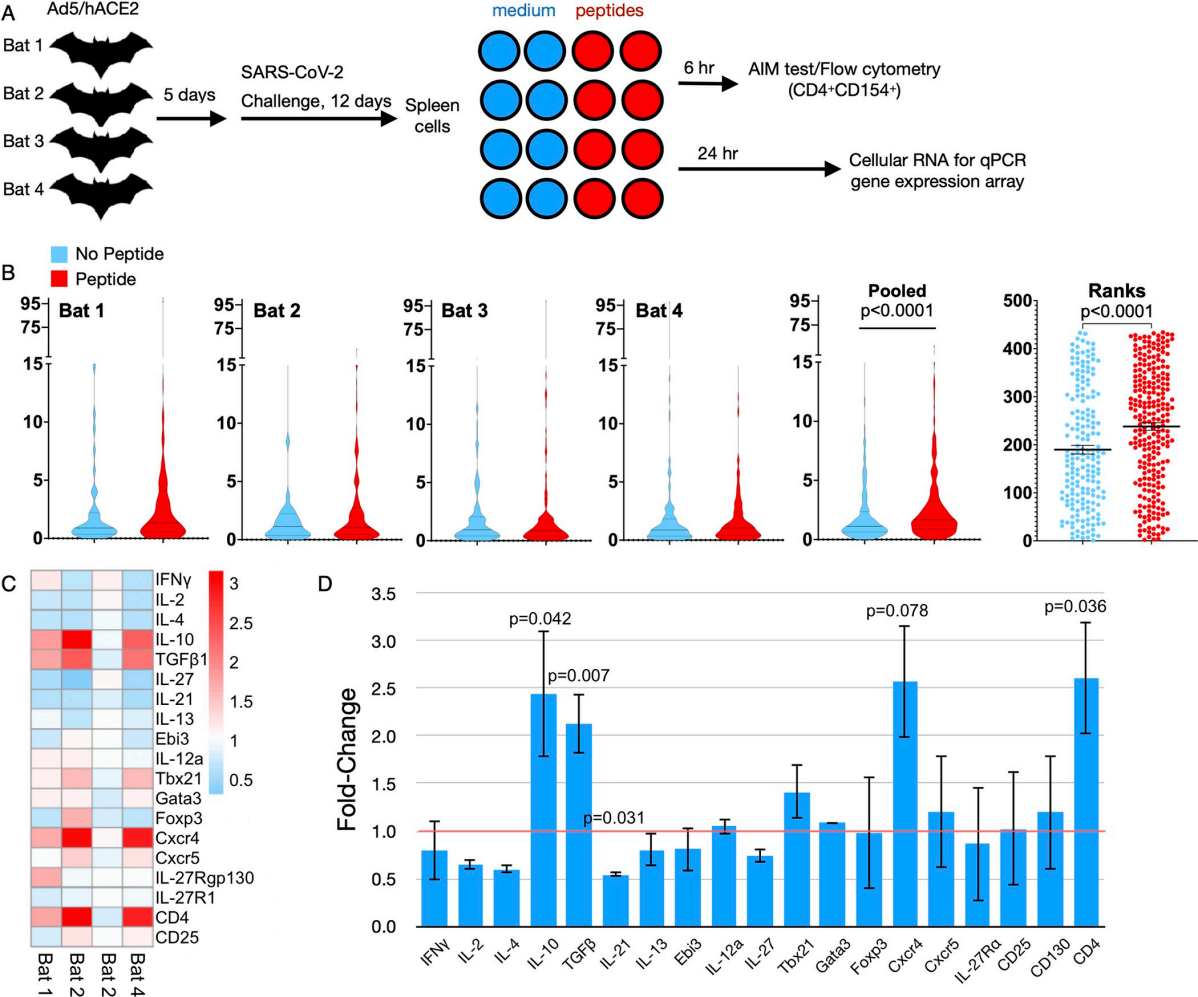
Activation of CD4^+^ Helper T cells in SARS-CoV-2-infected Jamaican fruit bats. Four bats were transduced with human ACE2 and infected 5 days later. After 12 days, splenocytes were recovered for AIM testing and gene expression analsysis. **A.** Splenocytes from hACE2-transduced bats 12 days post infection with SARS-CoV were cultured with or without SARS-CoV-2 nucleocapsid peptide library for 6 hours in the presence of anti-CD40 blocking antibody (AIM testing) or 24 hours without anti-CD40 blocking antibody (cellular RNA). **B.** Cells were stained with anti-CD154 and anti-CD4 and analyzed by flow cytometry. Violin plots for the individual bats, plus the pooled sample and rank data. In the presence of peptide, CD154 expression was significantly increased on splenocytes (red) compared to no peptide controls (blue) (Wilcoxon rank sum test). **C.** Heat map of splenocyte qPCR (ΔΔCq) gene expression in response to peptide stimulation determined 3 of the 4 bats expressed a profile consistent with a regulatory T cell response. **D.** Peptide-induced gene expression changes in splenocyte cultures of bats 1, 2 and 4 from panel C; IL-10, TGFβ and CD4 gene expression were significantly elevated, whereas CXCR4 was nearly significant (two-tailed t-test).

Four bats transduced with Ad5/hACE2 and challenged with SARS-CoV-2 were euthanized 12 days later (Fig 6A). Splenocytes were cultured with or without SARS-CoV-2 nucleocapsid peptide library and anti-CD40 blocking antibody in duplicate for 6 hours. The cells were then co-stained for CD4 and CD154. CD4^+^ CD154^+^ splenocytes were gated as follows: Intact Lymphocytes > Single Cells > Live Cells > CD4^+^ > CD154^+^. CD4 and CD154 gates were drawn using florescent minus one controls (FMOs) (S5 Fig). CVS files of CD4^+^CD154^+^ cells were then exported from FlowJo to obtain fluorescent intensities of CD154 on each cell for statistical analysis in Graph Pad Prism 9 (S5 Fig), and evaluated for CD154 expression by flow cytometry. The FSC and SSC profile of CD4^+^ CD154^+^ T cells support that these are lymphocytes (S6 Fig). Less than 2% of the CD4^+^ splenocytes also expressed CD154 (S5 Fig), which was significantly elevated on these cells that were stimulated by peptide (p<0.0001) compared to paired samples without peptide stimulation (Fig 6B). Replicate cells from the bats were cultured 24 hours with or without peptide library and RNA extracted for RT-qPCR for T cell gene expression profiling. Three of the four bats had increased expression of several genes; however, bat 3 did not exhibit changes in gene expression (Fig 6C). This bat also had minimal CD154 expression (Fig 6B), suggesting it was not responsive to antigen. Excluding this bat, IL-10 (2.44-fold, p = 0.042), TGFβ (2.12-fold, p = 0.007), and CD4 (2.61-fold, p = 0.036) expression were significantly elevated compared to splenocytes incubated without peptide library, and CXCR4 was nearly significant (2.58-fold, p = 0.078). IL-21 expression was significantly downregulated (1.82-fold, p = 0.031), and IL-2 (1.54-fold, p = 0.108) and IL-4 (1.67-fold, p = 0.174) expression were substantially, but not signficiantly, downregulated compared to splenocytes cultured without peptide library (Fig 6D).

## Discussion

A significant limitation for studying coronavirus infections of bats is the lack of captive colonies of relevant bat species and the availability of coronaviruses that are naturally hosted by those species. Sarbecoviruses are naturally hosted by insectivorous horseshoe bats (*Rhinolophus* spp.) and there are more than 80 species in the genus. Until such colonies are established, research on bats and coronaviruses will require surrogate bat models, similar to how the laboratory mouse is routinely used as a surrogate model. We established a breeding colony of Jamaican fruit bats in 2006 and have used bats from this colony for a number of infection studies with several viruses, including MERS-CoV [14,35–37].

The initial challenge study determined that SARS-CoV-2 replicated poorly in Jamaican fruit bats for only a few days without visible signs of disease, is confined to the intestine with detection of virus in the Peyer’s patches and mononuclear cells of the intestinal lamina propria, no apparent shedding in feces, and without a detectable antibody response, even by highly sensitive ELISA to nucleocapsid antigen. The lack of detectable virus in the lungs is consistent with what is known about coronavirus circulation in wild bat populations, where virus is typically found in the intestines and shed through feces. One possible explanation for this is that very low levels of endogenous ACE2 mRNA was detected in Jamaican fruit bat lungs. The only other experimental SARS-CoV-2 challenges of bats have yielded varying results. An experimental challenge of Egyptian fruit bats led to a limited infection without visible signs of disease, but with detectable virus in several tissues, including lungs and lymph nodes, and transmission to one contact bat [29]. Challenge of big brown bats did not lead to infection [30]; however, two studies of Mexican/Brazilian free-tail bats were contradictory, with one suggesting they were susceptible but not the other [31,32]. ACE2 residues that are thought to be important in SARS-CoV-2 spike binding [38,39] are more conserved in Egyptian fruit bats (16/20 residues with human), substantially less in big brown bats and Mexican/Brazilian free-tailed bats (11/20 residues), with Jamaican fruit bats intermediate (13/20 residues) (S1 Table). The conservation of these ACE2 residues varies substantially among several horseshoe bat species; thus, it may be that certain horseshoe bat species may favor evolution of sarbecoviruses that are more likely to infect humans based upon similarity of ACE2 contact residues. If so, then such conservation may be predictive of which horseshoe bat species should be monitored for sarbecoviruses with spillover potential. Currently, only 13 horseshoe bat species ACE2 sequences are available in GenBank, which is a fraction of the number species in the genus. Field studies to determine sequences of ACE2 from other horseshoe bats may be useful for identifying potential sarbecoviruses with pandemic potential.

It may be that SARS-CoV-2 spike protein, which mediates cellular attachment to ACE2, is substantially different compared to its ancestral virus because of the likely route of passage through one or more intermediate bridge hosts before spillover into humans, and subsequent adaptations as it transmitted among humans. As SARS-CoV-2 has continued to circulate among humans, new variants that have emerged are likely to be more divergent from the ancestral virus, such that SARS-CoV-2 may now be more of a “human virus” than a “bat virus.” Ideally, the original bat virus that spilled over into the bridge host(s) is required to fully understand the biology of the virus. However, it is unlikely the virus will ever be found because by now it may have gone extinct or has undergone substantial recombination with other sarbecoviruses, a common occurrence of coronaviruses [1,13], such that it no longer exists in nature. The earliest SARS-CoV-2 isolate available to us was WA1, which was isolated from a patient in Washington state, USA. It may be that earlier isolates, such as the initial Wuhan isolates, behave differently in Jamaican fruit bats. The results presented here suggest that (i) SARS-CoV-2 has limited ability to bind Jamaican fruit bat ACE2 (AjACE2) (poor susceptibility), which is consistent with a report demonstrating spike binding to AjACE2 [40]; (ii) it is unable to replicate in Jamaican fruit bat cells (poor permissibility); (iii) the innate response controls infection; or (iv) a combination of these features.

Several adenovirus serotypes use the coxsackievirus and adenovirus receptor (CAR, *Cxadr*) for cellular entry. We determined that Jamaican fruit bat CAR protein shares 86% identity and 92% similarity with the laboratory house mouse ortholog, suggesting it could be used for adenovirus transduction of human ACE2. To address this possibility, we adopted an approach previously developed for the assessment of SARS-CoV-1 and SARS-CoV-2 infection of laboratory mice, in which a replication-defective serotype 5 adenovirus (Ad5), which uses CAR for cellular entry, was generated that expresses human ACE2 (Ad5/hACE2) [33,34,41,42]. Upon transduction of Jamaican fruit bat primary kidney epithelial (Ajk) cells with Ad5/hACE2/eGFP, sustained fluorescence was observed for several days. However, inoculation of the cells with SARS-CoV-2 did not lead to detectable virus replication, suggesting the cells are not permissive. It is also possible that the cells lacked a suitable protease, such as TMPRSS2, that can facilitate SARS-CoV-2 penetration of the plasma membrane, or that adenovirus transduction activated antiviral pathways in the cells that restricted SARS-CoV-2 replication. Alternatively, SARS-CoV-2 can infect certain cells, such as Vero E6, via endosomal entry where cathepsin L facilitates endosomal escape of the genomic RNA into the cytoplasm [43]. A recent study determined that cell lines from several insectivorous bat species were not permissive to SARS-CoV-2 replication despite expression of endogenous bat ACE2 on some of these cells. One of the cell lines tested was from a patagium biopsy of a greater horseshoe bat (*Rhinolophus ferrumequinum*), a member of the genus that harbors sarbecoviruses [1]; however, it was unclear whether these cells expressed endogenous ACE2, or whether a lack of sufficient RfACE2 binding residues may impair spike binding (12/20 residues, S1 Table), and attempts to transduce this cell line with human ACE2 failed [44]. Transduction of human ACE2 led to low-level, nonproductive SARS-CoV-2 infection in four of the other bat cell lines, but none were of kidney origin like Ajk cells. One of the lines appeared to produce virus particles; however, electron microscopy indicated the viruses were unable to complete exocytosis. The authors postulated that tetherin, which appears to be under strong selective pressure in bats [45], may account for this observation. In the other cells, expression of *Oas1* and *Ifih1* (MDA-5) transcripts were elevated during SARS-CoV-2 infection and may have impaired virus replication.

Despite the discouraging results of the Ajk cells, we proceeded with hACE2 transduction of cells in the lungs of Jamaican fruit bats to determine susceptibility. Transduced bats challenged with 10^5^ TCID_50_ equivalents of SARS-CoV-2 WA1 did not show visible signs of disease and they did not lose weight (Fig 3D). BALB/c mice similarly transduced with hACE2 and challenged with SARS-CoV-2 exhibited significant weight loss of 10–25% during the first week but recovered thereafter [33]. It possible that the detected virus (Fig 3B) represents residual input virus; Hassan et al. detected low levels of viral RNA in the lungs of SARS-CoV-2 challenged BALB/c mice that had not been transduced with hACE2 [33]. Regardless, clear pathology was present in hACE-transduced and infected bat lungs (Fig 4A), antigen was readily detected in the main stem bronchi of challenged bats for up to 14 days, and clear but low-titer antibody responses occurred beginning on day 7 (Figs 3C and S1). This is consistent with work by us and others showing that bats typically produce low-titer antibody responses to viral infections [29,35,37,46–50]. We cannot exclude the possibility that adenovirus transduction elicited an innate response that could have augmented control of SARS-CoV-2 infection; however, mouse studies have shown innate stimulation from transduction subsides within a day of inoculation with the vector [51].

Total cellular quantification conducted in the bronchial epithelium and parenchyma revealed significant decreases in the amount of total cellular populations at 4 DPI and 10 DPI that recovered to baseline at 21 DPI in animals that were transduced with hACE2 adenovirus and subsequently challenged with SARS-CoV-2 (Fig 5). Notably, SARS-CoV-2 infection in other animal models and reports of clinical cases showed compelling histopathological evidence that SARS-CoV-2 infection leads to immune cell infiltration of the lung parenchyma and collapse of bronchial airways, evident through loss of bronchial epithelium [52–55]. The recruitment of immune cells to the lung in SARS-CoV-2 infection can occur as early as 2 DPI and is often progressive and results in permeant lung damage, severe disease, fibrosis, and/or death [54]. However, the reduction in total cellular populations within the lung parenchyma in concert with the retention of bronchial epithelium in bats transduced with hACE2 and then challenged with SARS-CoV-2 shows that these bats become susceptible to viral infection within the lungs only if hACE2 is present. Despite this, the apparent pathology does not appear to be as severe as in other mammalian model systems or clinical patients [52–55]. These results provide compelling evidence that although expression of hACE2 in bats allowed infection, the immune response of the bats likely mitigated severe pathological outcome and hypercellularity within the lungs. We are unable to determine if the moderate cellular destruction in the bat lungs is a result of direct viral infection, or whether it is caused by immune cells (e.g., CTL, NK). Monoclonal antibodies to markers found on these cells will be required to determine what events are occurring in the tissues.

We also determined that CD4^+^ helper T cells responded to SARS-CoV-2 infection in the hACE2 bats. Antigen-reactive T cells typically are rare in infected animals, usually fewer than 2% of T cells even days or weeks after infection, making assessment of ex vivo T cell responses challenging. However, because CD154 (CD40L) is an activation marker of helper T cells, its expression can be used to identify the rare helper T cells activated by antigen by flow cytometry [56,57]. We found a significant increase in CD154 expression on CD4^+^ helper T cells when stimulated with a SARS-CoV-2 nucleocapsid peptide library (Fig 6B). In humans, abundant CD4^+^CD154^+^ T cells are associated with severe COVID-19, particularly in T cells producing abundant proinflammatory cytokines, including interferon-γ (IFNγ), tumor necrosis factor, interleukin-2 (IL-2) and/or interleukin-21 (IL-21) [58,59]. In the splenocytes of 3 of 4 bats cultured 24 hours with nucleocapsid peptide library antigen, IL-10 and TGFβ expression were significantly elevated more than two-fold (Fig 5C and 5D) despite the low frequency (<2%) of antigen-specific helper T cells in the splenocyte cultures, whereas IL-21 transcripts were significantly repressed. IL-10 and TGFβ are typically produced by regulatory T cell subsets that suppress inflammatory responses [60], whereas IL-21 is expressed by T follicular helper (Tfh) cells and facilitates lymphatic germinal center formation and B cell class switching and affinity maturation [61–63]. Expression of Th1 IL-2 and IFNγ transcripts and Th2 IL-4 and IL-13 transcripts trended lower than basal level expression. Fox-p3 and CXCR5 expression were not substantially elevated, which could be explained by low levels of expression of this transcription factor and cell surface receptor, respectively, in the few antigen-reactive T cells (i.e., low signal-to-noise ratios). Identification or development of monoclonal antibodies for these bat orthologs could enhance their detection using flow cytometry, particularly to determine whether the Treg-like cells are expressing both IL-10 and TGFβ, or if there are two populations expressing each cytokine. Transcripts of helper T cell markers CD4 and CXCR4, which is substantially elevated in patients with severe COVID-19 [64], were both elevated, suggesting that CXCR4 activity may be less in bats compared to humans, or it may have a different role in bats. The study also determined that Treg cell responses during COVID-19 may lead to more severe outcomes in humans, which is seemingly contradictory considering IFNγ- and IL-21-expressing T cells appeared to contribute to immunopathology [59]. Thus, there appear to be similar responses, yet clear differences in helper T cell activities or subsets in bats and humans infected with SARS-CoV-2 in which Treg cells may limit inflammation in bats such that no meaningful disease occurs. The T cells from bat 3 did not have elevated expression of any gene in the qPCR array (Fig 5C). Repeated testing of the duplicate samples from this bat yielded similar results; thus, we are confident the RNA was properly extracted. It is also possible that the nucleocapsid peptide library was not added when the splenocytes were plated; however, because each T cell culture from each bat was performed in duplicate we think this is unlikely because replicates from both had similar Ct values. It may be that other genes not on the qPCR array were expressed by these T cells; RNA-Seq could clarify this question.

CD4^+^ helper T cells are instrumental for many effector functions and they provide the highest level of command and control of adaptive immune responses. Upon in vitro engagement between a CD4^+^ helper T cell receptor (TCR) and antigen presenting cell MHC class II/peptide complex, CD154 is transiently expressed on the T cell surface for about 6 hours, then declines. During this time, it can be assessed by flow cytometry as an activation maker and a surrogate for TCR/MHC/peptide engagement. Indeed, engagement of Th cell CD154 with B cell CD40 leads to T cell help, including IL-21 production from Tfh cells in germinal centers [63], for class switching and somatic hypermutation/affinity maturation. In the present study, we only detected low titer antibody responses in the bats, even at 21 days post infection, and the reduction of IL-21 expression may be one reason why antibody titers, which are principally a function of affinity maturation, are poor. In our previous experimental infection studies of Tacaribe virus-infected Jamaican fruit bats, which causes a fatal disease, RNA-Seq analysis identified genes required for somatic hypermutation in spleens, including activation-induced cytidine deaminase; however, they were not differentially expressed in infected bats [36]. Thus, it may be that bats have such robust innate immune responses that they are less dependent on antibody responses. However, prior to the present work, no studies have been published describing virus-specific helper or cytotoxic T cells in bats, principally because of a lack of tools for the study of bat adaptive immunity; thus, it is unclear what roles bat T cells play in immunity to viruses. It may be that bats have a greater dependency on regulatory T cells that could temper inflammatory pathology, which is consistent with the observation that bats typically have low or no inflammatory responses during viral infections [35,46,65,66]. This also parallels what occurs in rodent reservoirs of hantaviruses, in which TGFβ-expressing regulatory T cells predominate to what are otherwise innocuous infections [67,68]. Further studies requiring the development of new reagents and methods for bats will be needed to address these questions, particularly studies for examining individual T cells (e.g., T cell culturing, flow cytometry, single-cell RNA-Seq, etc.).

There are limitations of this study that should be addressed in future research, principally that this is a surrogate animal model that likely does not faithfully recapitulate the biology of sarbecovirus infections of their natural reservoir hosts. Viruses are likely fine-tuned to their reservoirs, thus the ideal sarbecovirus model would be one that uses the virus’ natural reservoir host horseshoe bat species. This would require obtaining a sarbecovirus or its complete genome, along with capture, quarantine and establishment of a SPF breeding colony of the reservoir bat species. One such model could be BANAL-236 CoV, which was isolated from a Marshall’s horseshoe bat (*Rhinolophus marshalli*) captured in Laos [13]. It is also important to note that tissue-specific immune responses can differ in natural reservoirs of viruses [69] and that mesenteric T cell responses in naturally-infected horseshoe bats may be substantially different than what occurs in the spleens because of the intestinal tropism of coronaviruses in bats. AIM tests have been developed for cytotoxic T lymphocytes by assessing elevated CD134 expression on CD8^+^ T cells [70]. However, we have been unable to identify a monoclonal antibody that recognizes Jamaican fruit bat CD8, thus one will need to be developed. Identification of cross-reactive antibodies to CD134 will also be necessary; otherwise, monoclonal antibodies to it will also need to be generated. It will be necessary to examine the kinetics of the T cell response using various time points and larger sample sizes for AIM testing and cytokine gene expression. Bats are of low fecundity and female Jamaican fruit bats give birth to one pup at 6-month intervals, limiting their availability for large sample size studies.

The findings here also show that serotype 5 adenovirus vectors are important tools for addressing viral infections of bats, including SARS-CoV-2 variants. It is likely that SARS-CoV-2 originated in horseshoe bats, thus it should be possible to address questions of SARS-CoV-2 and other sarbecovirus susceptibility and infection of Jamaican fruit bats transduced with ACE2 from horseshoe bat species. Although work with cells expressing various bat ACE2 proteins have been performed [40], a better understanding of how these viruses behave in bats, particularly adaptive immune responses which require live bats, could shed light on the biology of coronaviruses. A limitation of adenovirus use is that gene transduction is confined to the respiratory tract and not the intestines, where natural bat coronaviruses are typically found. Nonetheless, this approach offers a means to more fully dissect the role of a variety of viral infections of bats and how the adaptive immune system responds to those viruses.

## Materials and methods

### Ethics statement

This study was performed with approval from the Colorado State University Institutional Animal Care and Use Committee (protocol 1787).

### Jamaican fruit bats

Bats of both sexes were used; however, more males than females were used (2:1) because of low fecundity (one pup at 6 month intervals for reproductive females). The bat colony is maintained in a free-flight facility; however, infected bats were confined to bird cages under BSL-3 containment upon challenge with SARS-CoV-2.

### Generation of anti-Jamaican fruit bat CD4 monoclonal antibody

The Jamaican fruit bat CD4 protein was submitted to Phyre2 molecular modeling server and the generated structure file provided several candidate solvent-accessible peptides. An extracellular membrane-proximal sequence from residues 236–277 (ENRKVSVVKTRQDRR) with an N-terminal cysteine was generated and covalently linked to KLH (Genscript). BALB/c mice were intraperitoneally-immunized with 25 μg of the KLH-peptide conjugate emulsified in incomplete Freund’s adjuvant (IFA) and boosted at 1 month intervals (in IFA) for 3 months. Sera from mice were reactive to Jamaican fruit bat splenocytes. A final boost in PBS was administered and 4 days later splenocytes were fused with Sp2/10 Ag14 cells (ATCC CRL-1581) and cultured overnight prior to concurrent cloning and HAT selection on methylcellulose plates (ClonaCell-HY, StemCell Technologies). After 14 days of incubation at 37°C, clones were picked for expansion. Supernatants were screened by flow cytometry to identify reactive monoclonal antibodies. Several were identified and one, 1-D5 was selected for fluorescent labeling.

### Identification of cross reactive anti-CD40 and anti-CD154 antibodies: In silico protein homology

Jamaican fruit bat CD40 (NCBI, XP_037020498) and CD154 (NCBI, XP_037012946) polypeptide sequences were submitted to Phyre2 molecular modeling server to generate PDB files [71]. The generated Jamaican fruit bat protein PDB structures, mouse CD40 (UniProt, P27512), and human CD154 (UniProt, P29965) PDB files were imported into Geneious Prime for the assessment of protein homology by Blocks Substitution Matrix 62 (BLOSUM62) alignment [72]. The three-dimensional structures were then annotated for the transmembrane domains and identical amino acid residues to identify putative conserved regions to which commercially-available monoclonal antibodies might bind to Jamaican fruit bat orthologs.

### Identification of cross reactive anti-CD40 and anti-CD154 antibodies: Flow cytometry

One naïve male Jamaican fruit bat was euthanized and a single-cell suspension of its spleen was generated by mechanical disassociation in cold 10 ml PBS with 5 mM EDTA by passage through a 70μm cell strainer. Splenocytes were then placed in a 15 ml conical tube and centrifuged at 4°C for 5 minutes at 350 g and supernatant was decanted. Splenocytes were then resuspended in cold 10 ml ammonium chloride solution (150mM NH_4_Cl, 10mM NaHCO_3_, 10mM EDTA in cell culture grade water) and incubated for 10 minutes on an orbital rotator to lyse red blood cells. The cells were washed and resuspended in 10 ml PBS, 5 mM EDTA splenocytes were then counted using a hemocytometer and trypan blue and resuspended in cryopreservation media (90% FBS, 10% DMSO) at 5x10^6^ cells/ml, and 1 ml aliquots dispensed into cryovials. Cryovials were then stored in -80°C for 2 days, then transferred to liquid nitrogen.

One cryovial was retrieved from liquid nitrogen, thawed in a 37°C water bath, transferred to 15 ml conical tube and washed with 10 ml PBS, 5 mM EDTA. 10^6^ cells were transferred to wells of a 96 well V-bottom plate for the unstained control. Splenocytes were washed and resuspended in 5 ml of a 1:5,000 dilution of primary amine viability dye (Ghost DyeTM Red 780) in PBS with 5 mM EDTA solution and incubated in the dark at 4°C for 30 minutes. Splenocytes were then centrifuged at 4°C for 5 minutes at 350 g and supernatant was decanted, then washed in 10 ml FACS buffer (PBS, 1% BSA, 0.5% sodium azide, 5 mM EDTA) centrifuged at 4°C for 5 minutes at 350 g and supernatant was decanted. Cells were then resuspended in FACS buffer, and 1 million splenocytes were added to 96 well v-bottom plate per test sample. Splenocytes were then centrifuged at 4°C for 3 minutes at 350 g and supernatant was decanted. Splenocytes were then resuspended in 100 μl Fc receptor blocker (Innovex, ready-to-use) and incubated for 30 minutes at 4°C in the dark. Splenocytes were then centrifuged at 4°C for 3 minutes at 350g and supernatant was decanted. Test samples were then resuspended in a 1:20 dilution of anti-mouse CD40 PE (Tonbo, clone FGK45), or anti-human CD154 PE (Tonbo, clone 5C8) in FACS buffer and incubated for 30 minutes at 4°C in the dark. Splenocytes were then centrifuged at 4°C for 5 minutes at 350 g and supernatant was decanted. Splenocytes were washed 2x with 150μl FACS buffer centrifuged at 4°C for 5 minutes at 350g and supernatant was decanted each time. Splenocytes were then resuspended in 100μl FACS buffer. A 4 laser Cytek Auora (16V-14B-10YG-8R) cytometer was used to acquire the FCS files. The unmixed FCS files were then analyzed using FlowJo 10.8.1.

### Initial SARS-CoV-2 challenge study

The WA1 isolate of SARS-CoV-2 was used for this study and was obtained from BEI Resources and passaged twice in Vero E6 cells to generate a stock. For the initial study, 18 bats were inoculated with 10^5^ TCID_50_ of virus while holding the bats in a contralateral position to ensure delivery to the trachea and the esophagus (i.e., lungs and GI tracts) with 10^5^ TCID_50_ equivalents per bat in 50 μl in sterile PBS. Oral and rectal swabs were collected from bats on days 2, 4, 7, 10, 14 and 21 post-inoculation (PI) for detection of virus by qPCR. Three naïve bats added 2 days later for contact transmission, and 3 uninoculated control bats were separately housed (total of 24 bats). On days 2, 4, 7, 10, 14 and 21, oral and rectal swabs were collected from all bats and three inoculated bats were euthanized on these days, whereas the 3 contact bats and negative control bats were euthanized on day 21.

### Virus detection

For virus isolation, frozen lungs and intestines were homogenized with a Tissue Lyser II in 10% FBS DMEM, centrifuged at 8,000g for 5 min, then passed through a 0.2 μm filter to remove bacterial contaminants. Log_10_ dilution series were made for each sample and added to replicate 96 wells of Vero E6 cells and scored for CPE 5 days later to calculate TCID_50_. For qPCR, RNA was extracted from frozen tissues (Qiagen RNEasy kit) and TCID_50_ equivalents determined using a SARS-CoV-2 E gene detection kit (Promega) based upon the Berlin primer/probe set [73].

### Adenovirus transduction of Jamaican fruit bat kidney cell line with human ACE2

A serotype 5 defective adenovirus encoding the human ACE2 gene (Ad5CMVhACE2) purchased from the University of Iowa Viral Vector Core was used for this study. The E1 gene is replaced with human ACE2, rendering the virus incapable of replication. For in vitro studies, Jamaican fruit bat Ajk cells were inoculated with 1 MOI of a GFP-expressing virus (Ad5CMVACE2-IRESeGFP) for 1 hour, followed by removal of inoculum and 2x washing in 2% FBS-DMEM. For SARS-CoV-2 challenge, cells were inoculated with virus without GFP (Ad5CMVhACE2) as above and at 48 hours inoculated with 0.1 MOI of SARS-CoV-2. Supernatants were collected at 1 hr and daily thereafter for titration on Vero E6 cells.

### SARS-CoV-2 challenge of Jamaican fruit bats transduced with hACE2

For in vivo experiments, 21 bats were intranasally inoculated with 2.5x10^8^ pfu in 50 μl of serum-free DMEM. SARS-CoV-2 challenge of 18 of these bats was performed 5 days later. The remaining 3 hACE2 transduced bats that were not challenged with SARS-CoV-2 were used as controls, with one bat euthanized on days 4, 10 and 21. Groups of hACE2/SARS-CoV-2-challenged bats were euthanized (four on days 2, 4, 7; two on days 10, 14, 21; Fig 3A schematic) for necropsy with portions of lungs and intestine collected and frozen for viral RNA and virus isolation, and the remaining tissues collected in buffered formalin.

### Histopathology and Immunohistochemistry

Tissues and carcasses with open abdomens were collected in 10% neutral buffered formalin for 3 days prior to removal from the BSL-3 and transferred to the Colorado State University Diagnostic Laboratories for trimming. Oral cavity, salivary glands, olfactory bulb, cerebrum, cerebellum, and brain stem were thoroughly inspected for gross lesions. Additionally, lungs, spleens, kidneys, large and small intestines, liver, gall bladder, stomach and bladder were collected. Decalcified skulls and visceral organs were processed, embedded in paraffin wax and 4–5 μm sections were stained with hematoxylin and eosin for blinded evaluation by the pathologist using a Nikon i80 microscope (Nikon Microscopy).

Sections were stained using ultraView universal alkaline phosphatase red detection kit. Heat-induced epitope retrieval was performed on a Leica Bond-III IHC automated stainer using Bond Epitope Retrieval solution for 20 minutes. Viral nucleocapsid antigen was detected with a purified rabbit polyclonal antibody. Labeling was performed on an automated staining platform. Fast Red was used as chromogen and slides were counterstained with hematoxylin. Immunoreactions were visualized by a single pathologist in a blinded fashion. In all cases, normal and reactive mouse brain sections incubated with primary antibodies was used as a positive immunohistochemical control. Negative controls were incubated in diluent consisting of Tris-buffered saline with carrier protein and homologous nonimmune sera. All sequential steps of the immunostaining procedure were performed on negative controls following incubation.

### Fluorescent microscopy

Paraffin embedded tissue sections were stained for SARS-CoV-2 nucleocapsid protein (1:500) using a Leica Bond RXm automated staining instrument following permeabilization using 0.01% Triton X diluted in Tris-buffered saline (TBS). Blocking was performed with 1% donkey serum diluted in TBS. Sections were stained for DAPI (Sigma) and mounted on glass coverslips in ProLong Gold Antifade mounting medium and stored at ambient temperature until imaging. Images were captured using an Olympus BX63 fluorescence microscope equipped with a motorized stage and Hamamatsu ORCA-flash 4.0 LT CCD camera. Images were collected and regions of interest quantified with Olympus cellSens software (v 1.18) using an Olympus X- line apochromat 10X (0.40 N.A.), 20X (0.8 N.A.) or 40X (0.95 N.A.) air objectives, or Uplan Flour X100 oil immersion (1.3 N.A.) objective.

### Automated high-throughput immunofluorescence staining and imaging of tissue sections

Paraffin embedded lung tissue was sectioned at 10 μm thickness and mounted onto poly-ionic slides (Colorado State University Diagnostic Laboratory). Tissue sections were deparaffinized and immunofluorescently labeled using a Leica Bond RXm automated robotic staining system. Antigen retrieval was performed by using Bond Epitope Retrieval Solution 2 for 20 minutes in conjunction with heat application. Sections were then incubated with primary antibodies diluted in 0.1% triton-X containing tris-buffered saline (TBS): rabbit anti-SARS-CoV-2 nucleocapsid protein (Novus Biologicals; 1:100) and goat anti-human angiotensin converting enzyme 2 (ACE2; R&D Systems; 1:500). Appropriate species-specific secondary antibodies conjugated to AlexaFluor555 and/or AlexaFluor647 were applied for fluorescent detection. Sections were stained with DAPI (Sigma, 1:5,000) and mounted on glass coverslips in ProLong Gold Antifade hard set mounting medium and stored at 4°C until time of imaging.

### Artificial intelligence neural network detection and quantification of total nuclei in whole tissue sections

Whole lung section montage images were acquired on a fully automated motorized stage scanning VS200 microscope (Evident, Waltham, MA USA) equipped with a Hamamatsu ORCA-Fusion camera (Hamamatsu Photonics, Shizuoka, Japan) and imaged with at 200X resolution using an extended apochromat X-line 20X air (0.8 N.A.) using Olympus CellSens (v.3.2). All slides were stained and imaged simultaneously using consistent exposure times and CCD gain and binning parameters to reduce variability in intensity measurements. Images were then imported into Visiopharm Software (v.2022.09.0.12417) and the immunofluorescence nuclear detection application was modified to accommodate intensity range and average area of cells within the lung sections. Regions of interest (ROIs) were generated around whole lung sections based on DAPI positive staining intensity, where total nuclei detected within the tissue was normalized to the total area of whole tissue to minimize variation observed between differing sizes of sectioned lung. Multi-group and time-point comparisons were analyzed by 2-way ANOVA with Bonferroni post hoc analysis for intra-group statistical analysis.

### Serum IgG ELISA

The SARS-CoV-2 spike (S) ectodomain containing the prefusion stabilizing hexapro mutations [74] and a mutated furin cleavage site was produced and purified from Expi293 cells as previously described [75]. The SARS-CoV-2 S protein was diluted to 0.003 mg/mL in PBS and used to coat 384-well Nunc Maxisorp plates (Thermo Fisher) overnight at room temperature. The plates were slapped dry and blocked for 1 hour at 37°C using Casein in PBS (Thermo Fisher). Following blocking, the plates were again slapped dry and sera from infected bats was added to the plates beginning at a 1:30 dilution in TBST followed by a 1:3 serial dilution thereafter. Plates were incubated for 1 hour at 37°C, slapped dry, and washed four times with TBST. Recombinant protein A/G conjugated to HRP (Thermo Fisher) diluted 1:500 in TBST was added each well and the plates were incubated for 1 hour at 37°C then washed four times with TBST. To measure binding titers, TMB Microwell Peroxidase (Seracare) was added to each well and, after 2 minutes, the reaction was quenched with 1 N HCl. The absorbance at 450 nm was measured using a BioTek Neo2 plate reader and analyzed in GraphPad Prism 9 with ED_50_ values being determined using a four-parameter logistic regression model. Two technical replicates were performed for each sample.

### Neutralization assays

VSV particles pseudotyped with the SARS-CoV-2 S protein harboring the D614G mutation were produced as previously described [76,77]. To perform the neutralization assays, 20,000 Vero-TMPRSS2 cells were seeded into each well of a 96-well plate and grown overnight until they reached approximately 80–90% confluency. Sera from infected bats were diluted in DMEM (Gibco) beginning at a 1:10 dilution followed by a 1:3 serial dilution thereafter. The diluted sera were then incubated for 30 minutes at room temperature with SARS-CoV-2 S pseudotyped VSV diluted 1:25 in DMEM along with an anti-VSV-G antibody (I1-mouse hybridoma supernatant diluted 1:25, from CRL-2700, ATCC) to block entry of any residual VSV G pseudotyped virus. The Vero-TMPRSS2 cells were washed three times with DMEM and the virus-sera mixture was added to the cells. Two hours after infection, an equal volume of DMEM supplemented with 20% FBS and 2% PenStrep was added to each well. The cells were incubated for 20–24 hours after which ONE-GloEX (Promega) was added to each well and the plates were incubated for 5 minutes. Luminescence values measured in relative light units (RLU) were recorded using a BioTek Neo2 plate reader. The values were normalized in GraphPad Prism 9 with the RLU values from uninfected cells to determine 0% infectivity and the RLU values from cells infected with pseudovirus only to determine 100% infectivity. ID50 values were determined from the normalized data using a [inhibitor] vs. normalized response–variable slope model. Neutralization assays were performed in duplicate and replicated with two distinct batches of pseudovirus.

### Activation-induced marker test

Four bats were transduced with hACE2 and 5 days later inoculated with SARS-CoV-2. Twelve days later, the bats were euthanized and spleens collected to make single cell suspensions. Red blood cells were lysed with ammonium chloride buffer and 10^6^ splenocytes from each bat were cultured with 1 μg of SARS-CoV-2 PepTivator® SARS-CoV-2 Prot_N peptide library (15mers with 12 residue overlaps, Miltenyi) for 6 hours in 5% FBS Clicks medium (Fujifilm/Irvine Scientific) and 1 μg of anti-mouse CD40 monoclonal antibody (FGK45, Tonbo/Cytek, to block CD154 ligation) at 37° C in duplicate. Identical wells were set up in duplicate without peptide library to provide baseline marker levels. Cells were collected, washed and stained with viability dye (Ghost Dye Violet 450, Tonbo/Cytek, 1:5,000 dilution). Cells were then washed and stained with anti-Jamaican fruit bat CD4 (1:100 dilution) and anti-human CD154 (5C8, Tonbo/Cytek, 1:20 dilution). Cells were fixed in paraformaldehyde and examined by flow cytometry. CD4^+^ CD154^+^ splenocytes were gated as follows: Intact Lymphocytes > Single Cells > Live Cells > CD4^+^ > CD154^+^. CD4 and CD154 gates were drawn using florescent minus one controls (FMOs) (S5 Fig). CVS files of CD4^+^CD154^+^ cells were then exported from FlowJo to obtain the mean fluorescent intensity (MFI) of CD154 was determined. MFI were evaluated between groups using Wilcoxon signed-rank test.

### Immune gene expression profiling

Identical cultures described in the AIM test were examined for immune gene expression. Splenocytes (5x10^6^) were cultured in 24 well plates with 5 μg of SARS-CoV-2 PepTivator® SARS-CoV-2 Prot_N peptide library for 24 hours. Identical cultures without peptide library were used to determine baseline gene expression. Duplicate splenocyte cultures were examined for changes in gene expression by SYBR Green qPCR array as previously described [22,68,78]. Briefly, total RNA was extracted from the cells (RNEasy kit, Qiagen) and cDNA produced (QuantiTech RT, Qiagen). Primers for Jamaican fruit bat genes (S2 Table) and qPCR was performed (QuantiTech SYBR Green, Qiagen). Within sample gene normalization was performed on Rps18 (ΔCq), and fold-chage determined by comparing each gene from the peptide-stimulated cells to the unstimulated cells from the same bat (ΔΔCq).

## Supporting information

S1 Fig Neutralizing antibody titers from hACE-tranduced Jamaican fruit bats. Only bat 64 produced antibodies that neutralized VSV pseudotype virus expressing SARS-CoV-2 spike.
(TIF)

S2 Fig (A) Predicted extracellular structure of Jamaican fruit bat CD4 with immunizing peptide in orange, and (B) flow cytometric staining of naive bat splenocytes with monoclonal antibody 1-D5.
(TIF)

S3 Fig Protein homology of Jamaican fruit bat, mouse, and human CD40 and CD154. CD40 protein structures mouse (top left) and Jamaican fruit bat (top right). CD154 protein structures human (bottom left) and Jamaican fruit bat (bottom right). Protein structures are represented with signal peptides removed and transmembrane domains (magenta) highlighted for orientation. Jamaican fruit bat CD40 and mouse CD40 protein alignment identified 90 identical extracellular sites (red) or 53.39% identical extracellular domains with a BLOSUM62 value of 71.3%. Jamaican fruit bat CD40 and mouse CD40 protein alignment identified 39 identical cytoplasmic sites (cyan) or 72% identical cytoplasmic domains with a BLOSUM62 value of 84%. Jamaican fruit bat CD154 and human CD154 protein alignment identified 190 identical extracellular sites (red) or 88.4% identical extracellular domains with a BLOSUM62 value of 92.1%. Jamaican fruit bat CD154 and mouse CD154 protein alignment identified 18 identical cytoplasmic sites (cyan) or 61.9% identical cytoplasmic domains with a BLOSUM62 value of 73%.
(TIF)

S4 Fig Anti-mouse CD40 (FGK45) and anti-human CD154 (5C8) PE conjugated antibody staining of Jamaican fruit bat splenocytes. Gating strategy: Intact > Live > Single Cells > AF- > PE^+^. Anti-mouse CD40 cross reactive antibody demonstrated 20.7% positive splenocytes. Anti-human CD154 cross reactive antibody demonstrated 1.69% positive splenocytes.
(TIF)

S5 Fig Gating strategy to obtain CD4+ CD154+ splenocytes. Concatenated no peptide samples and FMOs (left) and concatenated peptide samples (right) were gated as follows: Intact Lymphocytes > Single Cells > Live Cells > CD4^+^ > CD154^+^. CVS files of CD4^+^CD154^+^ cells were then exported from FlowJo to obtain fluorescent intensities of CD154 on each cell for statistical analysis in Graph Pad Prism 9.
(TIF)

S6 Fig Forward (FSC) and side (SSC) scatter profiles of CD4+CD154+ cells (red dots) overlayed on all events (black dots) of concatenated peptide and concatenated no peptide flow cytometry data files. (TIF)

S1 Table Alignment of 20 ACE2 residues important for SARS-CoV-2 spike binding (cyan). Residues identical to human are noted in black, whereas differences in red font. Species that are susceptible to SARS-CoV-2 are in red font, not susceptible in blue font, unknown susceptibility in black font.
(XLSX)

S2 Table Immune gene expression primers, 5’ to 3’. (XLSX)
