# Supplementary figures and images for: Regulatory T cell-like response to SARS-CoV-2 in Jamaican fruit bats (Artibeus jamaicensis) transduced with human ACE2
Source: PLoS Pathog. 2023 Oct 19;19(10):e1011728. doi: 10.1371/journal.ppat.1011728 (PMC10617724; doi:10.1371/journal.ppat.1011728)

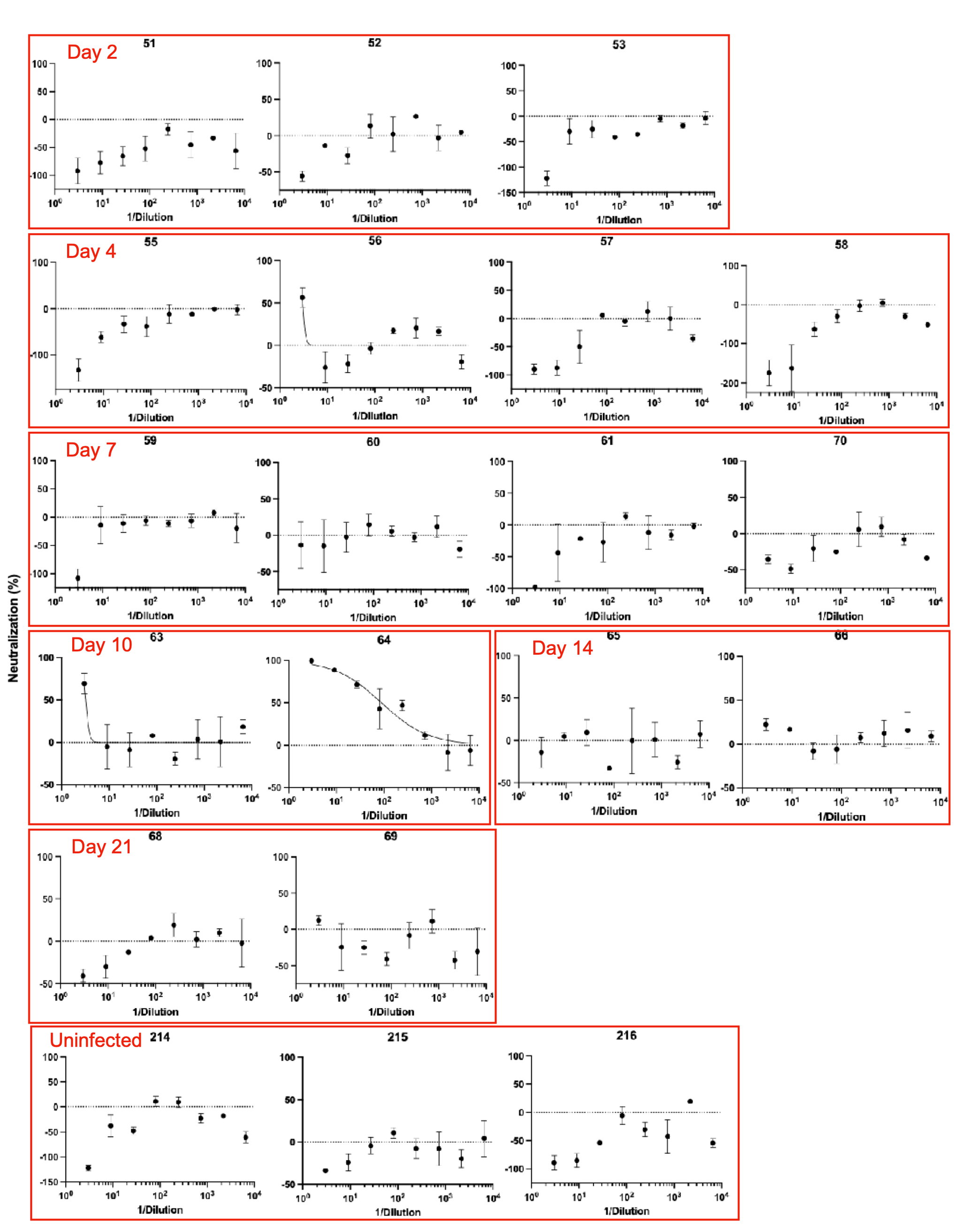

Supplement: S1 Fig — Only bat 64 produced antibodies that neutralized VSV pseudotype virus expressing SARS-CoV-2 spike. (TIF) [file ppat.1011728.s001.tif]

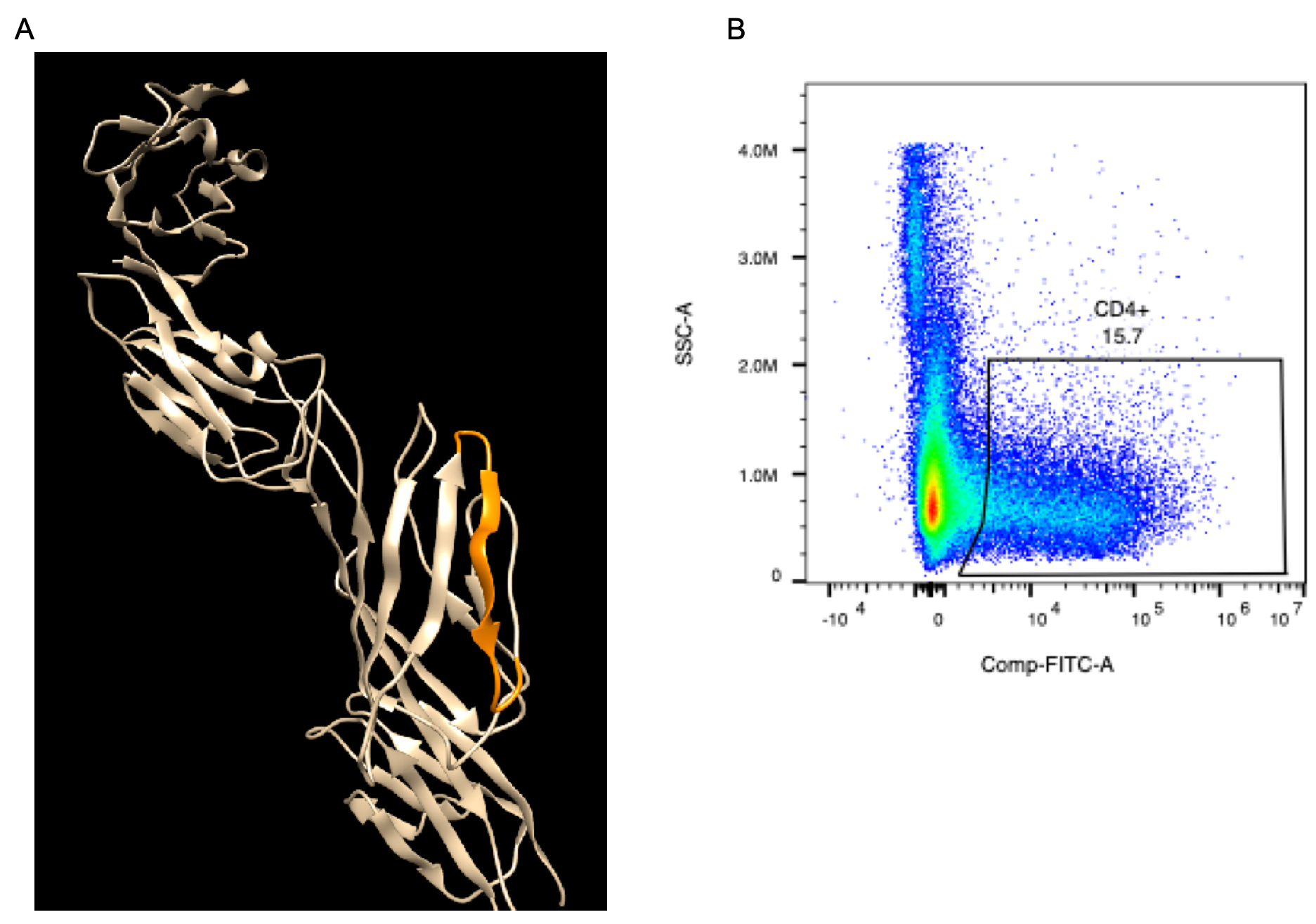

Supplement: S2 Fig — (A) Predicted extracellular structure of Jamaican fruit bat CD4 with immunizing peptide in orange, and (B) flow cytometric staining of naive bat splenocytes with monoclonal antibody 1-D5. (TIF) [file ppat.1011728.s002.tif]

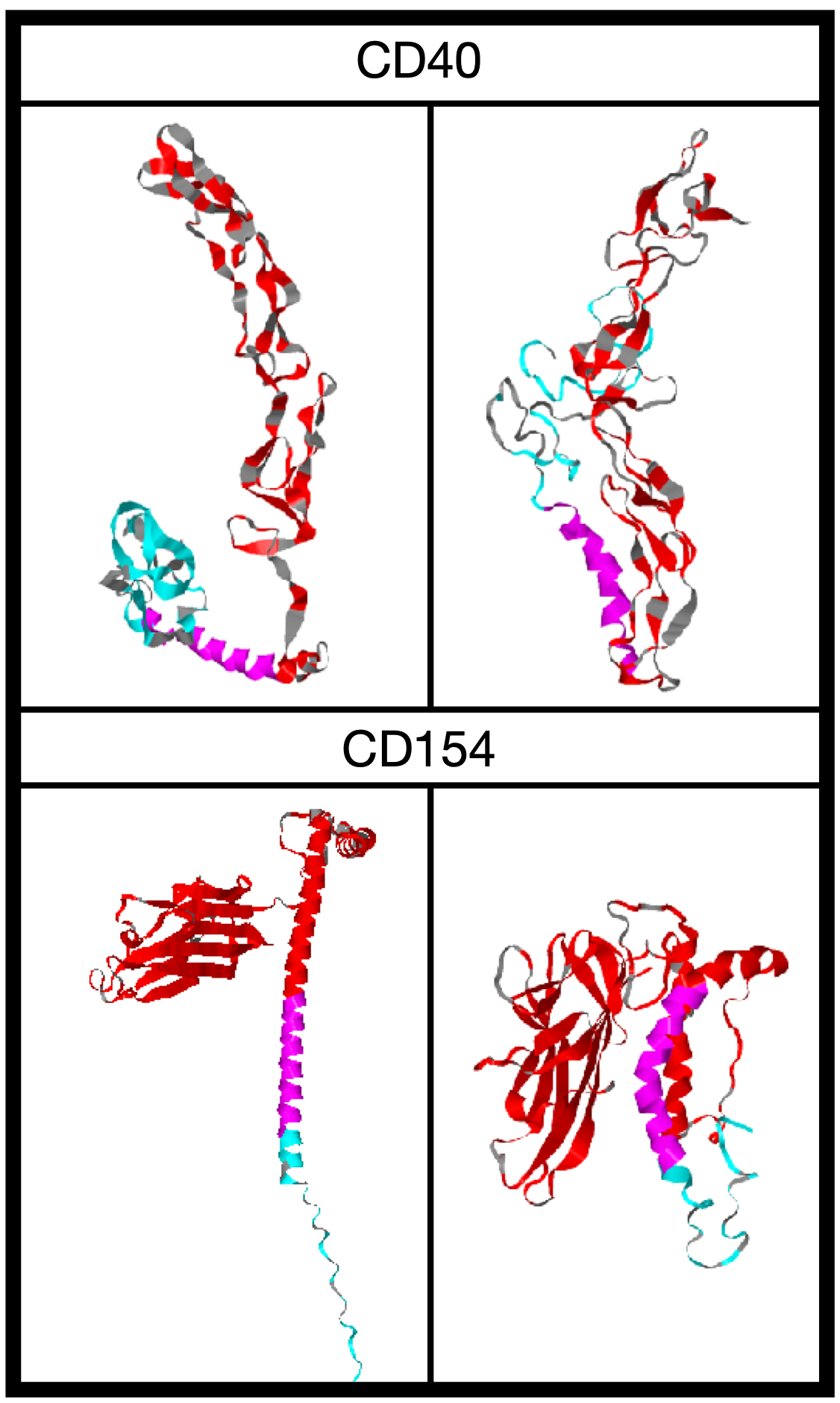

Supplement: S3 Fig — CD40 protein structures mouse (top left) and Jamaican fruit bat (top right). CD154 protein structures human (bottom left) and Jamaican fruit bat (bottom right). Protein structures are represented with signal peptides removed and transmembrane domains (magenta) highlighted for orientation. Jamaican fruit bat CD40 and mouse CD40 protein alignment identified 90 identical extracellular sites (red) or 53.39% identical extracellular domains with a BLOSUM62 value of 71.3%. Jamaican fruit bat CD40 and mouse CD40 protein alignment identified 39 identical cytoplasmic sites (cyan) or 72% identical cytoplasmic domains with a BLOSUM62 value of 84%. Jamaican fruit bat CD154 and human CD154 protein alignment identified 190 identical extracellular sites (red) or 88.4% identical extracellular domains with a BLOSUM62 value of 92.1%. Jamaican fruit bat CD154 and mouse CD154 protein alignment identified 18 identical cytoplasmic sites (cyan) or 61.9% identical cytoplasmic domains with a BLOSUM62 value of 73%. (TIF) [file ppat.1011728.s003.tif]

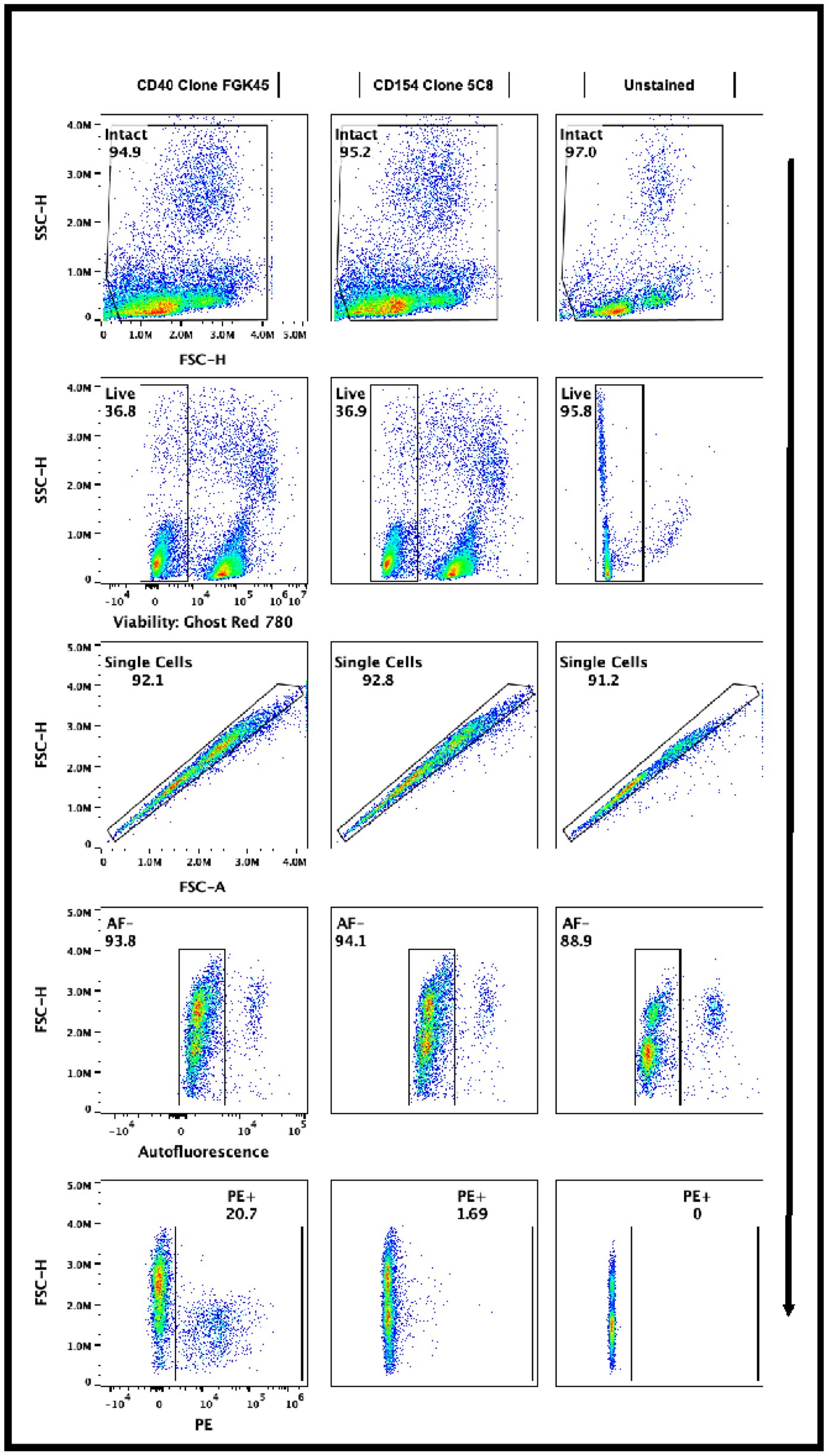

Supplement: S4 Fig — Gating strategy: Intact > Live > Single Cells > AF- > PE+. Anti-mouse CD40 cross reactive antibody demonstrated 20.7% positive splenocytes. Anti-human CD154 cross reactive antibody demonstrated 1.69% positive splenocytes. (TIF) [file ppat.1011728.s004.tif]

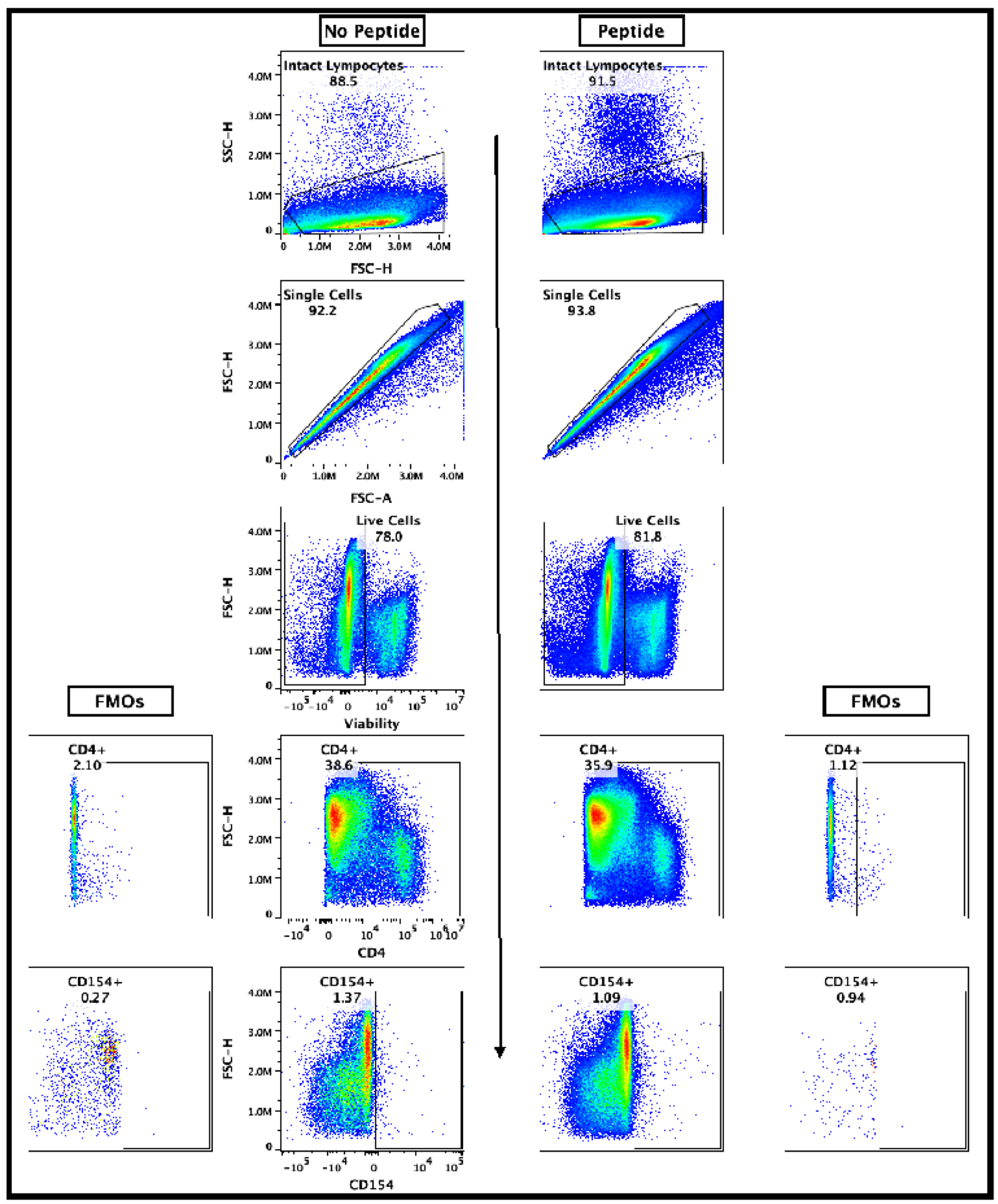

Supplement: S5 Fig — Concatenated no peptide samples and FMOs (left) and concatenated peptide samples (right) were gated as follows: Intact Lymphocytes > Single Cells > Live Cells > CD4+ > CD154+. CVS files of CD4+CD154+ cells were then exported from FlowJo to obtain fluorescent intensities of CD154 on each cell for statistical analysis in Graph Pad Prism 9. (TIF) [file ppat.1011728.s005.tif]

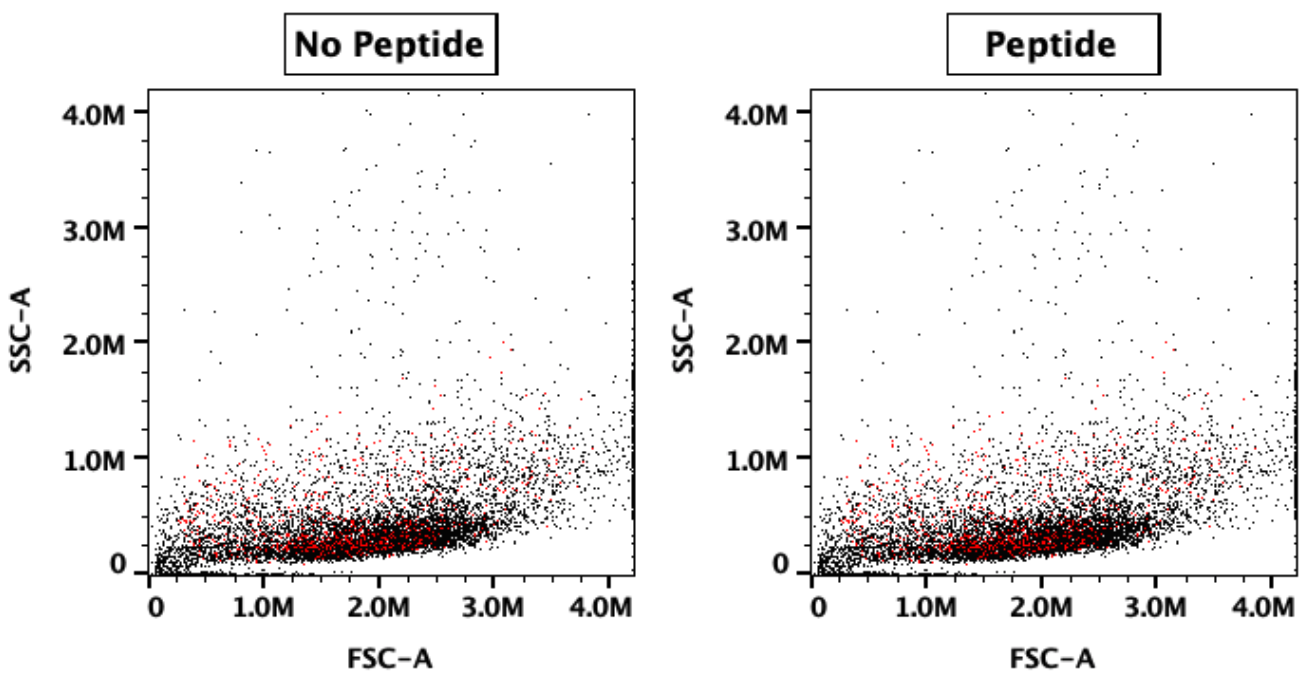

Supplement: S6 Fig — (TIF) [file ppat.1011728.s006.tif]
